# Engineered B7‐H3 Binding in Modular Gp2 Miniproteins

**DOI:** 10.1002/bit.70221

**Published:** 2026-04-29

**Authors:** Abbigael Harthorn, Hannah K. Windsor, Zachary Schmitz, Nathaniel Cheung, Ebube Agwaramgbo, Benjamin J. Hackel

**Affiliations:** ^1^ Department of Biomedical Engineering University of Minnesota – Twin Cities Minneapolis MN USA; ^2^ Department of Chemical Engineering and Materials Science University of Minnesota – Twin Cities Minneapolis MN USA

**Keywords:** affinity maturation, B7‐H3, directed evolution, Gp2, ligand, miniprotein, stability

## Abstract

B7‐H3, an important immune checkpoint modulator of T‐cell function, is a tumor vasculature biomarker and is overexpressed in a variety of cancers. Its expression is associated with tumor growth, metastasis, and poor clinical prognosis, which makes B7‐H3 an appealing target for diagnostics and therapeutics. High‐affinity, specific, modular ligands are needed to achieve the various modes of molecular targeting strategies. A designed combinatorial library of the small (45 amino acid) Gp2 scaffold was sorted for binders to B7‐H3 via yeast surface display with magnetic and flow cytometric cell sorting. Select variants were sequenced, characterized for binding affinity and specificity to B7‐H3, and assessed for modularity in a protein‐protein fusion. Protein ligands achieved single‐digit nanomolar affinities and retained binding affinity upon conjugation, via a glycine‐rich linker, to an enzyme. Directed evolution resulted in a potent, 0.7 nM binder with increased stability as assessed through the apparent midpoint of denaturation of 67°C. The engineered ligands provide small, modular, high‐affinity B7‐H3 binders for molecularly targeted therapeutics and diagnostics.

## 1 Introduction

B7‐H3, also known as CD276, is an important immune checkpoint modulator of T‐cell function (Picarda et al. 2016). It is also a tumor vasculature biomarker overexpressed in a variety of cancers, including clear cell renal cell carcinoma (Crispen et al. 2008), cutaneous melanoma (Wang et al. 2013), diffuse intrinsic pontine glioma (Zhou et al. 2013), hypopharyngeal squamous cell carcinoma (Katayama et al. 2011), non‐small cell lung cancer (Sun et al. 2006), ovarian cancer (Lutz et al. 2014), prostate cancer (Yuan et al. 2011), and pancreatic cancer (Yamato et al. 2009). Its expression is associated with tumor growth and metastasis and ultimately leads to poor clinical prognosis. The significant role of B7‐H3 in cancer progression and its differential expression in tumor vasculature make B7‐H3 an appealing target for therapeutic and diagnostic applications (Zhao et al. 2022; Guo et al. 2023).

Specific, high‐affinity binders are needed for such molecular targeting applications. While antibodies are typically used, certain properties hinder select applications. Large size (e.g., 150 kDa immunoglobulin G) limits solid tumor targeting via poor extravasation from vasculature and poor penetration through tissue (Jain 1994; Thurber et al. 2007, 2008). Large size and Fc receptor binding slow plasma clearance, resulting in high background, thereby hindering diagnostic imaging (Natarajan et al. 2013; Lu et al. 2020; Luo et al. 2022) and highly potent drug delivery (e.g., radiotherapy (Ninatti et al. 2025; To et al. 2025) or immune engagement (Tufail et al. 2025)). The molecular complexity of antibodies – disulfide bonds, glycosylation, and multi‐domain structures (Lee and Jeong 2015) – typically necessitates mammalian cell production and complicates the engineering of conjugated multifunctional formats (Gera 2022; Sawant et al. 2020). Thus, alternative binding scaffolds have been developed in attempts to address these shortcomings. Gp2, a 45‐amino acid truncated form of T7 phage gene 2 protein, has been evolved to bind numerous targets with high affinity while maintaining stability and modularity (Kruziki et al. 2015, 2018, 2016).

In this work, B7‐H3‐binding Gp2 ligands were engineered via yeast surface display, using magnetic and flow cytometric cell sorting to select for high‐affinity, specific, and stable variants from designed combinatorial libraries. Select variants were sequenced, characterized for binding affinity to B7‐H3, and assessed for modularity as a protein fusion. Gp2 ligands achieved single‐digit nanomolar binding affinities and retained binding affinity when genetically fused to an enzyme via a glycine‐rich linker. Directed evolution resulted in further maturation to subnanomolar potency (K_d_ = 0.7 nM) while elevating thermal stability (T_m_ = 67°C). The engineered ligands provide small, modular, high‐affinity B7‐H3 binders for molecularly targeted therapeutics and diagnostics.

## 2 Results

### 2.1 Engineered Protein Scaffolds Demonstrate High‐Affinity Binding to B7‐H3

**Figure 1:**
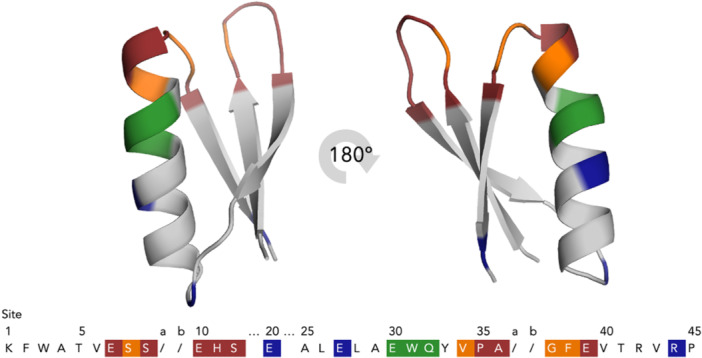
Gp2 Library Design. Gp2 (PDB 2WNM) was diversified across multiple sublibraries. A 1st‐generation library (Kruziki et al. 2015) permitted all 20 amino acids at each site within the two loops (red and orange). A mixture of 2nd generation libraries introduced sitewise constraint (detailed in (Kruziki et al. 2018)): Residues in red were designed to have up to all 20 amino acids across most libraries. Residues in orange were moderately constrained in multiple libraries while still encoding up to all 20 amino acids in other libraries. Residues in green were conserved with the exception of moderate diversification in one library. Residues in blue encoded wild‐type or a single neutral homolog.

**Figure 2:**
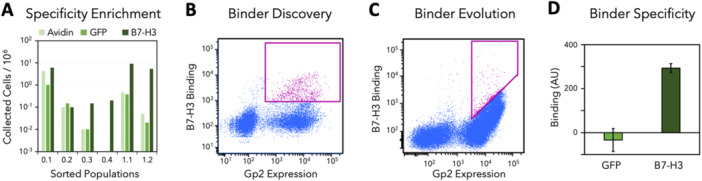
Enriched binders to B7‐H3. (A) Sorting progress. Yeast displaying Gp2 variants underwent three MACS selections in series (to generate populations 0.1‐0.3), followed by FACS (0.4). The resultant population was mutated via error‐prone PCR and further sorted via two MACS selections (1.1 and 1.2). Enrichment of B7‐H3‐specific binders is apparent as populations are further sorted. Binding to control avidin and negative GFP were not analyzed in the FACS sort. (B) Following the three MACS selections, yeast B7‐H3 binding was quantified by incubation with 50 nM biotin‐labeled recombinant human B7‐H3 extracellular domain, followed by streptavidin‐AlexaFluor 647. Scaffold expression was quantified with mouse anti‐c‐Myc antibody and goat anti‐mouse‐AlexaFluor 488. The gate collected c‐Myc^+^ variants with binding signal in the highest 4%. Flow cytometric analysis of 10,000 random variants is shown. (C) The enriched population was labeled with 1 nM biotin‐B7‐H3 cell lysate, streptavidin‐AlexaFluor647, and chicken anti‐c‐Myc‐FITC. The top 0.7% of c‐Myc^+^ binders were collected. (D) The final evolved population, after sorting with 1 nM B7‐H3 lysate, was yeast‐displayed and labeled with 100 nM biotin‐GFP or biotin‐B7‐H3 followed by streptavidin‐AlexaFluor 488 and quantified by flow cytometry. Data are presented as the mean ± standard deviation of the mean background‐subtracted (0 nM) median signal of two replicates.

We sought to engineer binders with strong affinity and specificity to B7‐H3. We used a Gp2 (Kruziki et al. 2015, 2018) library, designed with sitewise constrained diversity (Figure 1) for an increased frequency of developable, functional binders. The library was sorted, using yeast surface display with magnetic‐activated cell sorting (MACS) and fluorescence‐activated cell sorting (FACS), to discover specific binders to B7‐H3. The library underwent three MACS selections to deplete non‐specific binders to beads coated in avidin or green fluorescent protein (GFP) and enrich binders to recombinant human B7‐H3 extracellular domain immobilized on magnetic beads. Tenfold selectivity for B7‐H3 relative to controls was observed upon three sorts (Figure 2A). FACS using 50 nM recombinant B7‐H3 protein enriched binders (Figure 2B).

DNA was isolated from the enriched population and randomly mutated via error‐prone PCR of, separately, the full Gp2 gene or the diversified loops. Upon electroporation back into yeast, the mutated populations underwent a multivalent MACS selection, followed by a monovalent MACS sort with 100 nM recombinant human B7‐H3. As recombinantly produced soluble ectodomains often yield binders to epitopes not present or accessible in the intact cellular format (Stern et al. 2019), the population was then further enriched with two FACS selections using detergent‐solubilized MS1‐B7‐H3 lysate. Following an initial sort with 50 nM B7‐H3, stringency was increased by reducing the B7‐H3 concentration to 1 nM. Strong binders were observed and collected (Figure 2C). The specificity of variants in the enriched populations was assessed by incubating yeast with 100 nM biotin‐GFP, a non‐target protein, which exhibited negligible binding as compared to strong binding to 100 nM B7‐H3 (Figure 2D).

**Figure 3:**
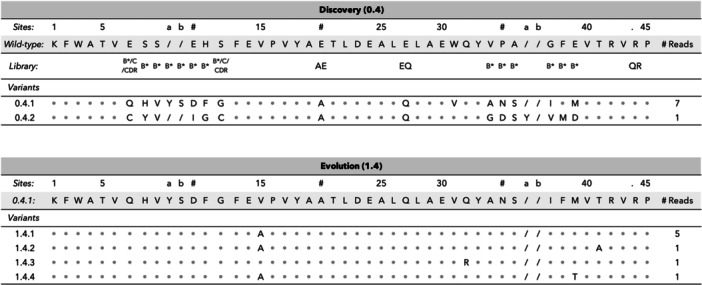
Sequences of discovered and evolved B7‐H3‐binding Gp2 ligands. Amino acid options are listed in the *Library* row. B* indicates all 20 amino acids biased toward amino acid options effective in ligand paratopes (Kruziki et al. 2018; Woldring et al. 2017a).

Single colonies of the evolved population were stochastically selected and sequenced at the discovery stage (four binding sorts from the naïve library without mutation [0.4]) and after the binding evolution stage (two MACS selections and two FACS lysate selections post‐error‐prone PCR [1.4]) (Figure 3). Initial binder discovery was predominantly led by one variant, Gp2_0.4.1_. Sequence analysis reveals that the lead variants most likely emerged from the sitewise‐constrained second‐generation Gp2 library that included an expanded paratope (resulting in the W31V mutation). Affinity evolution yielded several point mutants of this lead variant, most frequently V15A in Gp2_1.4.1_.

### 2.2 Protein Scaffolds Exhibit Modular, High‐Affinity Binding

**Figure 4:**
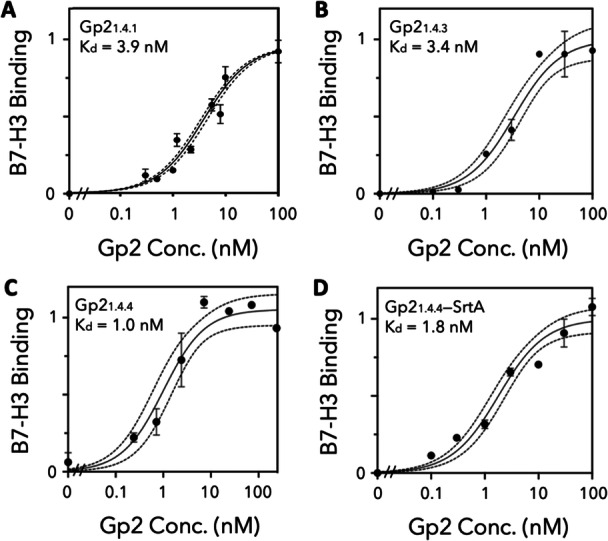
Affinity characterization of B7‐H3‐binding protein ligands and fusions. Purified Gp2 proteins were incubated with MS1‐B7‐H3 cells at the indicated concentrations. Binding was detected with FITC‐conjugated anti‐His_6_ antibody via flow cytometry. The best‐fit estimate of K_d_ and 68% confidence interval are indicated by solid and dashed lines, respectively. Data are presented as mean ± standard error with at least two replicates for each concentration. (A) Gp2_1.4.1_, (B) Gp2_1.4.3_, (C) Gp2_1.4.4_, and (D) Gp2_1.4.4_‐SrtA.

Affinity titration of recombinantly produced variants Gp2_1.4.1_, Gp2_1.4.3_, and Gp2_1.4.4_ yielded dissociation constants of 3.9 nM (68% confidence interval: (3.4–4.5 nM), 3.4 nM (2.7–4.2 nM), and 1.0 nM (0.8–1.3 nM) (Figure 4A–C), respectively. Single‐digit nanomolar affinities are consistent with strong physiological targeting (Okarvi and Jammaz 2012; Zahnd et al. 2010; Adams et al. 2001; Schmidt and Wittrup 2009; Thurber and Wittrup 2008; Orcutt et al. 2012).

**Figure 5:**
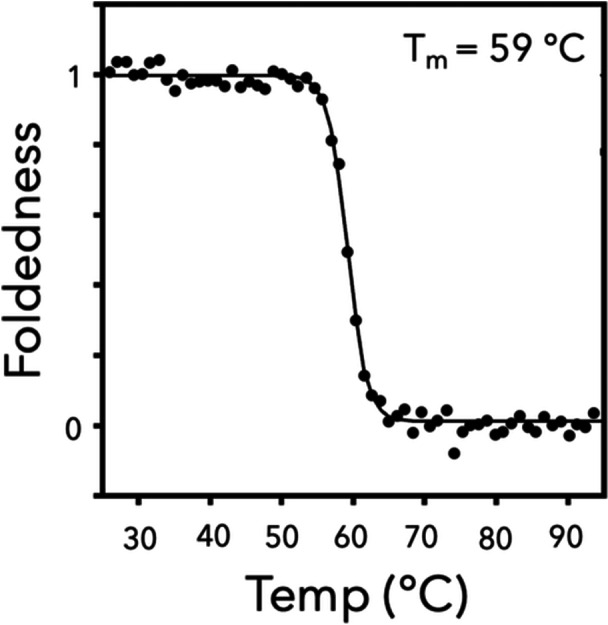
Stability of Gp2_1.4.1._ The apparent midpoint of thermal denaturation (T_m_) for Gp2_1.4.1_ was determined via CD spectroscopy. Molar ellipticity was monitored at 218 nm while the temperature was ramped from 25°C to 95°C.

The most abundant variant, Gp2_1.4.1_, was also evaluated for thermal stability via circular dichroism. The apparent midpoint of thermal denaturation, T_m_, was 59°C (95% confidence interval, 58°C–60°C; Figure 5).

Targeted proteins are often conjugated to therapeutic payloads, diagnostic moieties, cellular engagers, or nanoparticles to provide multifunctionality (Tian et al. 2022; Jin et al. 2022; Wu et al. 2022). Yet, proteins often have weakened function when conjugated (Zhao et al. 2008; Amet et al. 2009; Bai and Shen 2006; Bai et al. 2005). Protein ligand modularity was assessed by the retention of B7‐H3 binding affinity of the highest affinity variant, Gp2_1.4.4_, upon C‐terminal fusion, via a glycine‐rich linker, to sortaseA enzyme. Ligand‐enzyme fusions have potential utility in targeted diagnostics and targeted prodrug therapy. More broadly, these fusions serve as an example of other ligand‐protein fusions for multifunctional applications. Affinity titration of Gp2_1.4.4_‐L_15_‐SrtA fusion resulted in a K_d_ of 1.8 nM (1.4–2.2 nM) (Figure 4D), comparable to Gp2_1.4.4_ alone. Consequently, Gp2 1.4.4 is modular in this context, retaining function when fused to another protein.

### 2.3 Engineering Improved Protein Ligand Affinity, Specificity, and Stability

**Figure 6:**
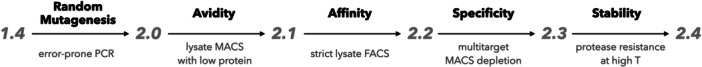
Sort scheme to improve affinity, specificity, and stability. Populations 2.1, 2.2, 2.3, and 2.4 were deep sequenced.

After characterization of lead variants, we strove to further improve the affinity as well as select for improved specificity and stability. The 1.4 population underwent random mutagenesis via error‐prone PCR. DNA was electroporated into yeast and further sorted (Figure 6).

**Figure 7:**
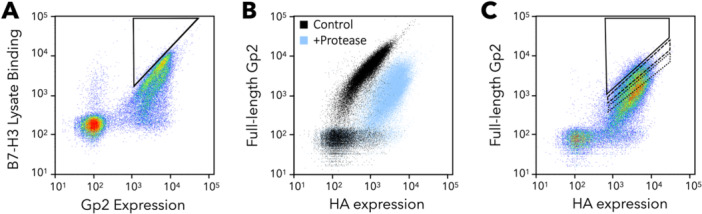
Selection for improved affinity and stability. (A) Yeast displaying the MACS‐sorted 2.1 population were incubated with ~0.2 nM biotinylated B7‐H3 cell lysate, labeled with chicken anti‐Myc‐FITC and streptavidin‐AlexaFluor647, and evaluated via flow cytometry. The strongest binders were isolated for further sorting. (B) After affinity selection and subsequent selectivity MACS, the 2.3 population was incubated with 0.75 mg/mL thermolysin at 55°C for 10 min and labeled with rabbit anti‐HA‐biotin and mouse anti‐Myc, followed by streptavidin‐AlexaFluor488 and goat anti‐mouse‐AlexaFluor647. Comparison of untreated (*Control*) and treated (*+Protease*) reveals the decrease in full‐length signal in the presence of thermolysin protease. (C) Yeast exhibiting the top 2%, next 14%, and next 19% of C‐terminal (Myc) to N‐terminal (HA) ratio were sorted.

Magnetic bead selection was performed with 20‐fold oversampling to select for B7‐H3 binders and deplete non‐binders resulting from mutagenesis. Enriched yeast was then incubated with ~0.2 nM biotinylated‐B7‐H3 cell lysate; numerous Gp2 variants with strong binding were observed, and the top variants were isolated via FACS (Figure 7A). To enrich highly specific binders, yeast underwent multitarget MACS depletion. Yeast was incubated with magnetic beads coated in a panel of non‐target proteins (lysozyme, bovine serum albumin, human interleukin receptor 2 gamma, TEV protease, human plasminogen activator urokinase receptor, human carbonic anhydrase II, and rabbit IgG‐FITC). Unbound yeast was collected and underwent a second negative depletion step to increase the depletion of non‐specific binders. The resultant Gp2 population was then sorted for protease stability using a yeast display assay (Golinski et al. 2021; Ritter and Hackel 2019; Klesmith et al. 2019). Yeast displaying Gp2 variants were treated with thermolysin at 55°C for 10 min, chilled, and labeled with antibodies for N‐ and C‐terminal epitopes. The population exhibited a range of susceptibility to protease – evidenced by varying C‐terminal:N‐terminal ratios resulting from proteolysis (Figure 7B) – thereby motivating stratification into stability tiers (Figure 7C).

**Figure 8:**
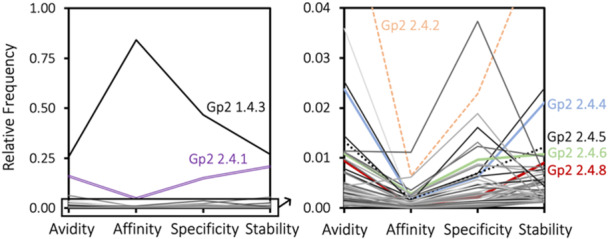
Relative frequency of variants across each sort: avidity, affinity, specificity, and stability (top gate). Sorting resulted in two very dominant variants: Gp2 1.4.3 and a new variant, Gp2 2.4.1. The zoomed‐in panel (right) shows the relative frequency of remaining variants. A handful of variants are labeled, and connecting lines are drawn for visual reference.

Gp2 populations after each sort were deep sequenced, and frequencies at each stage were computed (Figure 8). The change in frequency (i.e., enrichment) of each variant from each selection reports on its efficacy; thus, comparison of frequencies across the sorts informs on affinity, specificity, and stability impact. An array of outcomes is observed (Figure 8).

The final population contained 576 unique variants, where the top 20 variants accounted for approximately 70% of the read count. The most frequent new variants are shown in Table 1. The most frequent Gp2 variant was Gp2_1.4.3_, even after depletion at subsequent specificity and stability selections (Figure 8). This is likely because the initial frequency of this variant was particularly high. Gp2_1.4.1_ ranked 38th most frequent in the final population, while Gp2_1.4.4_ ranked 365th. Gp2_1.4.2_ was not observed in the final population. Despite wild‐type V15 and mutant Q32R within the predominant Gp2_1.4.3_, the remainder of the top variants have a V15A mutation and wild‐type Q32, as in the predominant variant in the 1.4 population (Gp2_1.4.1_). Of all sequences, no variant had positive enrichment at all affinity, specificity, and stability sorts. Numerous variants were heavily depleted in the affinity selection but largely enriched following specificity and stability sorts (Figure 8). Only 33 variants in the final population had positive enrichment post‐affinity sort (Affinity > 1); however, the mean final frequency of these variants was 0.00032 ± 0.00056, indicating these variants were severely depleted in the stability sort or largely infrequent.

**Table 1:** Lead Gp2 ligands determined from relative frequency after final sort.

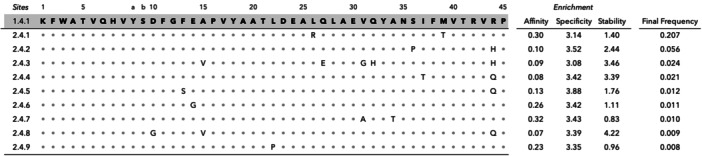

*Note:* The nine most abundant variants, aside from Gp2_1.4.3_, are presented. Sequences are aligned relative to variant Gp2_1.4.1_. The enrichments at each sort (affinity, specificity, and stability) were determined as the ratio of variant frequencies post‐sort to pre‐sort via deep sequencing. The final frequency is also presented.

#### 2.3.1 Sequences of Lead Gp2 Ligands Following Second Mutagenesis

**Figure 9:**
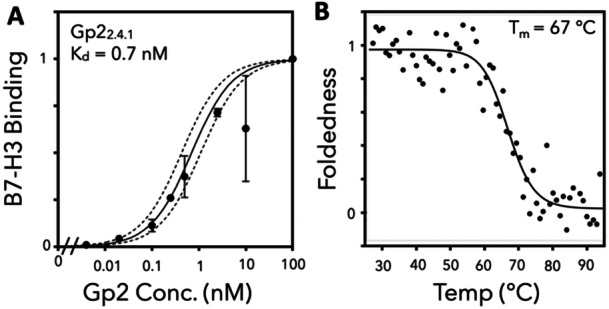
Affinity and stability characterization of Gp2_2.4.1_. (A) Purified Gp2_2.4.1_ was incubated with MS1‐B7‐H3 cells at the indicated concentrations. Binding was detected with FITC‐conjugated anti‐His_6_ antibody via flow cytometry. The best‐fit estimate of K_d_ and 68% confidence interval are indicated by solid and dashed lines, respectively. Data are presented as mean ± standard error of three replicates for each concentration. (B) The apparent midpoint of thermal denaturation (T_m_) for Gp2_2.4.1_ was determined via CD spectroscopy. Molar ellipticity was monitored at 218 nm while temperature was ramped from 25°C to 95°C.

From the final stability sort, the second most dominant clone to Gp2_1.4.3_ was a novel sequence, Gp2_2.4.1_ (Table 1, Figure 8), and thus subsequently characterized for affinity and stability. Affinity titration of Gp2_2.4.1_ resulted in a K_d_ of 0.7 nM (0.4–1.1 nM) (Figure 9A). The stability of Gp2_2.4.1_ was evaluated through the T_m_ and found to be 67°C (95% confidence interval, 64°C–69°C; Figure 9B), which is an increase of 8°C relative to Gp2_1.4.1_ (Kruziki et al. 2015, 2018).

### 2.4 Structural Analysis of High Affinity Gp2 Variants

**Figure 10:**
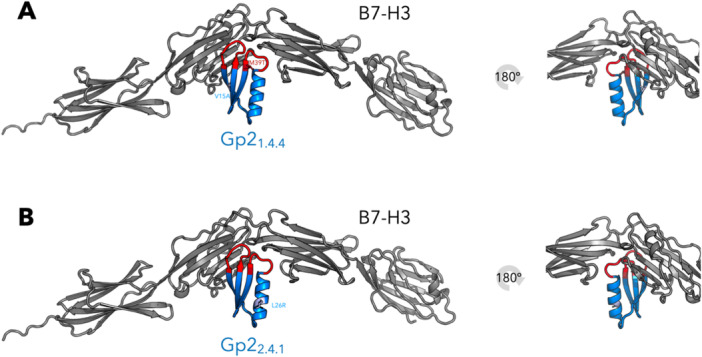
Structural analysis of high‐affinity Gp2 ligands. AlphaFold3 predicted interface between the extracellular domain of B7‐H3 (gray) and Gp2_1.4.4_ (A) or Gp2_2.4.1_ (B) Gp2 ligands (blue) have loop diversifications highlighted in dark red, and mutations stemming from error‐prone PCR in light blue. Unique residues for either Gp2_1.4.4_ or Gp2_2.4.1_ have side chains shown.

The highest affinity clones, Gp2_1.4.4_ and Gp2_2.4.1_ are predicted, via AlphaFold3 (Abramson et al. 2024), to engage with B7‐H3 in the same manner through putative engagement of the heavily diversified loop regions to B7‐H3 (Figure 10, dark red). It is important to note that while AlphaFold3 has demonstrated relatively high accuracy in dual‐protein complex docking, the resultant structural models are imperfect; while runs were initiated from numerous seeds to identify the most confident model, subsequent experiments will be needed to validate the proposed structures. AlphaFold3 iPTM scores were in the *confident* range (0.76 and 0.75 for B7‐H3 with Gp2_1.4.4_ and Gp2_2.4.1_, respectively) with the least confidence in the precise structure of the linker between the second and third domains of B7‐H3, the Gp2 loops, and the C‐terminal portion of the B7‐H3 extracellular domain (Figure S1). The top poses from five independent seeds are closely aligned for Gp2_1.4.4_ (template modeling (TM) scores = 0.87–0.98 [mean: 0.95], Figure S2A) and Gp2_2.4.1_ (0.96–0.99 [mean: 0.97], Figure S2B).

## 3 Discussion

In this study, miniprotein binders to cancer biomarker B7‐H3 were engineered using the Gp2 scaffold. A collection of Gp2 libraries was stringently sorted with MACS and FACS, resulting in binders with single‐digit nanomolar affinity. Lead variants emerged from the library with extensive sitewise amino acid constraints, which support the benefit of reducing detrimental amino acid options and enriching preferential options with a designed *de novo* discovery library (Woldring et al. 2015a, 2017; Hackel et al. 2010; Fellouse et al. 2007; Binz et al. 2004; Koide et al. 2012), as well as an expanded paratope, which supports the benefit of diversifying secondary structure beyond solvent‐exposed loops (Blanchard et al. 2023). The strongest lead (K_d_ = 1.0 nM) maintained strong binding upon fusion to sortase enzyme, exemplifying its modularity for multifunctional applications. Directed evolution via random mutagenesis for affinity and stability maturation yielded a variant with subnanomolar affinity (K_d_ = 0.7 nM) and elevated thermal stability (T_m_ = 67°C), consistent with approved biologics (Jain et al. 2017). There is consistent support via AlphaFold3 structural analysis that the miniproteins bind, primarily via their engineered loops, near the interface of the second and third Ig domains of B7‐H3, yet experimental validation is needed, particularly for precise atomic orientation. The engineered Gp2 miniproteins provide small (45 amino acids), high‐affinity, stable ligands amenable to multiple B7‐H3 targeting applications.

## 4 Materials and Methods

### 4.1 Library Growth and Selection Overview

The Gp2 library, detailed in Kruziki et al. (2015, 2018), comprised a truncated form of T7 phage gene 2 protein, diversified at six sites in each of two loops using degenerate codons encoding for an amino acid distribution mimicking antibody CDRs (Kruziki et al. 2015), as well as designs that constrained diversity and extended the paratope (Kruziki et al. 2018). Yeast surface display (Boder and Wittrup 1997; Chao et al. 2006) was used to present the Gp2 library as a C‐terminal fusion to Aga2p on the surface of EBY100 *S. cerevisiae* yeast from a pCT expression vector. Yeast were grown in synthetic media with dextrose and casamino acid media (SD‐CAA; 16.8 g/L sodium citrate dihydrate, 3.9 g/L citric acid, 20 g/L dextrose, 6.7 g/L yeast nitrogen base, 5 g/L casamino acids) at 30°C, 250 rpm, and display was induced with synthetic media with galactose casamino acids (SG‐CAA; 0.1 M sodium phosphate, 19 g/L galactose, 1 g/L dextrose, 6.7 g/L yeast nitrogen base, 5 g/L casamino acids, pH 6.0) at 30°C, 250 rpm.

B7‐H3 binders were selected using both MACS and FACS. In particular, the library was sorted three times via MACS to deplete non‐specific binders and enrich binders to multivalent recombinant B7‐H3. The population was then sorted via FACS for binders to 50 nM recombinant B7‐H3. DNA was then isolated from yeast and subject to random mutagenesis via error‐prone polymerase chain reaction (PCR) of either the paratope alone or the entire gene. DNA was electroporated back into yeast for display and further sorted via one multivalent MACS, one monovalent MACS, and subsequent FACS with detergent‐solubilized lysate from B7‐H3 expressing cells to ensure binding in the cellular context. Experimental details follow.

### 4.2 MACS Selections With Soluble Extracellular Domains

Magnetic bead selections were carried out, essentially as previously described (Ackerman et al. 2009), using at least 15‐fold oversampling of ligand diversity at all stages. Biotinylated recombinant human B7‐H3 extracellular domain (Sino Biological, catalog: 11188‐H08H‐B) for positive isolation and biotinylated Renilla reniformis green fluorescent protein (rrGFP; Avidity, cat: rrGFP‐100) for depletion were incubated with Dynabeads Biotin Binder (Invitrogen, cat: 11047) to coat the beads with protein. Yeast underwent MACS, where induced yeast were incubated with control bare biotin binder beads for 2 h at 4°C, followed by another 2‐h incubation for depletion with GFP‐labeled beads, to remove any non‐specific and non‐B7‐H3 binding interactions. Yeast was then incubated for 2 h more with beads with immobilized recombinant human B7‐H3 target protein. Bound yeast was collected. MACS was performed at 4°C, and the yeast was washed twice between incubations. A total of three MACS selections were performed with increasing wash stringency, where yeast were grown and induced between each sort. The enriched Gp2 population was sorted via FACS with recombinant B7‐H3 (detailed below). The resultant Gp2 genes were then mutated via error‐prone PCR, detailed below. The mutated population was subjected to an additional MACS sort. Collected yeast underwent a more stringent monovalent MACS sort. Yeast were incubated with control bare biotin binder beads, washed, and non‐specific binders or GFP binders were depleted by GFP‐coated beads, as before. Yeast were then washed and incubated with 100 nM soluble biotinylated recombinant human B7‐H3 extracellular domain for 1 h. Bare Biotin Binder beads were spiked in to bind to biotinylated B7‐H3 and were incubated for an additional 2 h at 4°C. Beads were washed three times, and the yeast remaining bound was collected.

### 4.3 Error‐Prone PCR

Random mutation was performed by error‐prone PCR with mutagenic analogs, 8‐oxo‐dGTP and dPTP (Zaccolo et al. 1996). Plasmid DNA was recovered from enriched yeast by zymoprep (Zymo Research). Plasmid DNA was mutated by error‐prone PCR of full Gp2 genes using primers W5 and W3 (Supplemental Table 1) and Gp2 loops using primers loop 1 and loop 2 (Kruziki et al. 2015). PCR products were purified by agarose gel electrophoresis. Loop gene fragments were assembled into one construct using PCR assembly. Final gene inserts were amplified by PCR, concentrated by ethanol precipitation, and resuspended for electroporation. Mutated libraries were homologously recombined with linearized pCT‐Gene (Chao et al. 2006) into EBY100 yeast by electroporation transformation (Woldring et al. 2015; Gietz et al. 1992; Gietz and Schiestl 2007). Electroporation yielded 33 million transformants.

### 4.4 FACS Selections With Soluble Extracellular Domains

After the three initial MACS sorts from the naïve Gp2 library, library size was efficiently sampleable via FACS. Induced yeast populations were simultaneously labeled with mouse anti‐c‐Myc antibody (9E10, BioLegend, Cat: 626802) and 50 nM biotinylated recombinant human B7‐H3 extracellular domain for 30 min at 4°C. Cells were washed once with phosphate‐buffered saline with 1 g/L bovine serum albumin (PBSA), labeled with goat anti‐mouse AlexaFluor 647 conjugate (Thermo Fisher Scientific, cat: A‐21235) and streptavidin AlexaFluor 488 conjugate (Thermo Fisher Scientific, cat: S‐11223) for 20 min at 4°C, and washed with PBSA. Cells that were Myc positive and had a B7‐H3 signal above background were collected.

### 4.5 FACS Selections With Detergent‐Solubilized Cell Lysates

After enrichment and mutation of binders and two subsequent MACS sorts detailed above, enriched populations underwent two rounds of flow cytometry selections with detergent‐solubilized cell lysate. Mile Sven 1 cells stably transfected to express human B7‐H3 (MS1‐B7‐H3) were grown at 37°C with 5% CO_2_ in DMEM with 10% fetal bovine serum (v/v) and 1% penicillin and streptomycin. MS1‐B7‐H3 cells were grown to 70–90% confluence in 75 cm^2^ tissue culture‐treated flasks. Cells were washed with phosphate‐buffered saline (PBS) and detached with trypsin‐EDTA treatment for 5 min, quenched with serum‐containing culture media, and pelleted at 500 g for 3 min. Pelleted cells were washed twice and resuspended in PBS with 0.5 mg/mL fresh sulfo‐NHS‐biotin (Thermo Fisher Scientific, cat: 21217) for 30 min at room temperature. Cells were washed twice following incubation to remove excess biotin and were lysed in 250 μL lysis buffer for 15 min at 4°C. Cell debris was pelleted for 30 min at 10,000 g and removed. Yeast was washed once with PBSA and incubated with cell lysate for 1 h at 4°C. Following incubation, yeast were washed, incubated with chicken anti‐Myc‐FITC (Immunology Consultants Laboratory Inc., cat: CMYC‐45F) and streptavidin, AlexaFluor 647 conjugate (Thermo Fisher Scientific, Cat: S21374) for 20 min at 4°C, and washed again. Yeast that were Myc positive (FITC) with the highest ratio of MS1‐B7‐H3 lysate binding (AlexaFluor647): Myc (FITC) were collected using FACS. This sort was repeated with higher stringency by using a lower B7‐H3 lysate concentration.

### 4.6 Sanger Sequencing of Yeast PCR Product

Enriched B7‐H3‐binding populations were plated on SD‐CAA plates and grown for 2 days. Ten colonies were stochastically chosen and incubated at 100°C for 5 min in 50 µL of distilled water. Two microliters of the boiled yeast sample were taken and underwent PCR with GeneAmp5/3 primers and DNA clean up. Amplified genes and GeneAmp5 primer were sent to Eurofins Genomics LLC for Sanger sequencing.

### 4.7 Cloning, Protein Expression, and Purification

Gp2 encoding regions in DNA recovered from the final B7‐H3 flow cytometry sort were amplified by PCR, digested with NheI‐HF and BamHI‐HF restriction enzymes (New England Biolabs), and ligated with T4 DNA ligase into pET‐24b vector containing a C‐terminal hexa‐histidine tag. Plasmids were transformed into T7 Express Competent *E. coli* and plated on lysogeny broth (LB) plates containing 50 mg/L kanamycin. Transformants were Sanger sequenced for full‐length gene and proper transformants were grown in 5 mL liquid LB with kanamycin (50 mg/L) at 37°C at 250 rpm for 12–16 h. Saturated cultures were added to 100 mL LB, grown, and induced. Cells were pelleted and resuspended in lysis buffer (50 mM sodium phosphate (pH 8.0), 0.5 M sodium chloride, 5% glycerol, 5 mM 3‐[(3‐cholamidopropyl) dimethylammonio]‐1‐propanesulfonate, and 25 mM imidazole), frozen and thawed five times to lyse cells, centrifuged for 10 min at 4°C, and 0.25 mm filtered. The resulting cell lysates were applied to 0.25 mL Cobalt HisPur resin volume spin columns, washed with 30 mM imidazole, and eluted with 300 mM imidazole. Purity and concentration were analyzed via protein gel electrophoresis with lysozyme standards.

For ligand‐enzyme fusions, the Gp2 gene encoding regions were amplified by PCR and assembled via NEBuilder HiFi DNA Assembly (New England Biolabs, cat: E2621S) into the pET‐24b vector containing a GSG_3_SG_3_KG_3_GT linker and SortaseA_60‐206_ (EC 3.4.22.70) gene (Supplemental Table 2) with a C‐terminal hexa‐histidine tag. Plasmids were transformed into BL21‐CodonPlus (DE3)‐RIL competent *E. coli* and plated on LB plates containing 50 mg/L kanamycin. Transformants were Sanger sequenced for the full‐length gene, and proper transformants were grown in 50 mg/L kanamycin and chloramphenicol for bacterial growth and protein purification. Purified proteins were affinity titrated to assess binding affinity in the presence of conjugated protein enzyme.

### 4.8 Affinity Titrations

Detached MS1‐B7‐H3 cells were washed and individually labeled with varying concentrations of purified Gp2 and Gp2 fusions for at least 30 min at 4°C. Cells were pelleted at 500 g for 3 min and washed with cold PBSA prior to labeling with anti‐His_6_ FITC conjugate (Abcam, cat: ab1206) for 20 min at 4°C. Fluorescence was analyzed using an Accuri C6 Plus. The dissociation constant was calculated by nonlinear least‐squares regression using a 1:1 binding model in GraphPad Prism with the exception of Gp2 2.4.1, which was fit under non‐equilibrium conditions. The k_off_ of Gp2 2.4.1 was fit using a k_on_ of 2.5×10^5^ M^‐1^s^‐1^ and a non‐equilibrium binding model by minimizing the sum of squared errors in GraphPad Prism 9.0.

### 4.9 Further Maturation of Affinity, Specificity, and Stability via Random Mutagenesis

DNA was isolated from yeast populations and subjected to random mutagenesis via error‐prone PCR (as described in the previous section) of the variable regions. DNA was electroporated back into yeast for display and further sorted for avidity, affinity, specificity, and stability.

B7‐H3 cell lysate MACS with decreased avidity: Magnetic bead selection was performed with 20‐fold oversampling to select for B7‐H3 binders and deplete non‐binders resulting from epPCR. Yeast was incubated with bare streptavidin‐coated beads for 2 h at 4°C, followed by another 2‐h incubation for depletion with GFP‐coated beads. Unbound yeast were incubated for 2 h with beads coated with 1 pmol of detergent‐solubilized biotinylated MS1‐B7‐H3 cell lysate. This is a 33‐fold decrease in the amount of antigen necessary to fully coat the beads and was done to increase stringency. Bound yeast was collected and grown.

Strict affinity FACS: Induced yeast populations were labeled with ~0.2 nM biotinylated‐B7‐H3 cell lysate for 2 h, washed, and labeled with streptavidin‐AlexaFluor647 and chicken anti‐Myc‐FITC. The top 0.4% of cells were collected and grown for the following sort.

Multitarget MACS depletion for increased specificity: To select for more specific binders, the population underwent a multitarget depletion. The following proteins were biotinylated, incubated with streptavidin‐coated beads, and used for negative depletion: lysozyme, bovine serum albumin, human interleukin receptor 2 gamma, TEV protease, human plasminogen activator urokinase receptor, human carbonic anhydrase II, and rabbit IgG‐FITC. These proteins were used because they were readily available and/or already conjugated to biotin. One million yeast cells were incubated with the aforementioned proteins coated on beads for 2 h at 4°C. Yeast then underwent the same depletion step a second time. Non‐binding yeast was selected and grown for the next sort.

Thermolysin and thermal stability FACS sort: To select for stable binders, the population underwent a protease incubation at increased temperature. Yeast was induced to display Gp2, incubated with 0.75 mg/mL thermolysin for 10 min at 55°C, then put on ice. Yeast were washed, incubated with mouse anti‐Myc and rabbit anti‐HA‐biotin for 20 min, washed, and labeled with goat anti‐mouse‐AlexaFluor647 and streptavidin‐FITC for another 20 min. Of the yeast that were HA + , those with the top 2%, 14%, and 19% of Myc:HA ratio were collected using FACS.

### 4.10 Library Preparation and Deep Sequencing Via Illumina iSeq

Plasmid DNA from sorted ligand populations (as depicted in Figure 6) was isolated from yeast using Zymolyase and extracted via silica spin column. The full Gp2 gene was amplified via PCR using the iSeq application primer. Illumina adapters were added in a second PCR to differentiate between populations, and DNA was quantified by Nanodrop and mixed in equal molar ratios for a final concentration of 5 nM. Samples were submitted to the University of Minnesota Genomics Center for quality control analysis and sequenced using Illumina iSeq (2 × 150 bp). Sequences were processed using USearch, filtered for any reads with > 0 N bases and a maximum of 1 total expected error for all bases in the read, and further filtered for full‐length Gp2 variants. Gp2 scaffold analysis was completed via homemade Python scripts to evaluate the frequency of unique variants and site enrichment of sorted populations.

The frequencies at each sort (e.g., avidity, affinity, specificity, and stability) were determined for each sequence, as well as their subsequent enrichment. In the stability sort, three‐tiered gates were collected (Figure 7C) and differentially sequenced. The read counts from each of the three stability gates were summed together for a total stability read count used to calculate one stability frequency for each sequence. To enable calculation of non‐infinite enrichment for variants that were not observed in the prior round, a read count of 1 was assigned for the prior round. The *z*‐score for affinity, specificity, stability, and final frequency was computed, and a final weighted average *z*‐score was calculated for all variants with affinity enrichment > 0.5 and final frequency > 0. An ‘enrichment’ of 0.5 (which is essentially two‐fold reduction) was chosen as a threshold to account for variants that were initially highly prevalent and thus had diminished opportunity to enrich. *Z*‐scores were averaged based on the following weights: affinity = 4, frequency = 3, specificity = 2, and stability = 1.

### 4.11 Circular Dichroism

The apparent midpoint of thermal denaturation (T_m, app_) was determined by circular dichroism (CD). Purified protein in 10 mM phosphate buffer (0.341 g/L sodium dihydrogen phosphate, 1.015 g/L sodium phosphate dibasic, pH 7.4) was diluted to ∼0.1 g/L in phosphate buffer, and the thermal stability and secondary structure were measured via CD using a JASCO J‐815 CD spectropolarimeter (Biophysical Technology Center, University of Minnesota). Thermal stability was measured by monitoring ellipticity at 218 nm as the temperature increased from 25°C to 95°C with a 3°C/min ramp. Melting temperatures were determined using two‐state unfolding equation and minimizing the sum of squared errors between predicted and empirical data (Hackel et al. 2008). Spectra were obtained by scanning from 260 to 190 nM at 25°C before thermal treatment.

### 4.12 AlphaFold Analysis

Default AlphaFold 3 (Abramson et al. 2024) (AF3) settings were utilized to predict engagement between the extracellular domain of B7‐H3 (Q5ZPR3, residues 1–456) and Gp2 variants. Five independent runs were performed with unique seeds. The top pose from each of the five runs was structurally aligned using the Protein Data Bank structural alignment tool with TM‐align (Bittrich et al. 2024).

## Author Contributions

A.H. led experimental work, data analysis, drafted the initial manuscript, and contributed to project conceptualization and manuscript editing. H.L. contributed to molecular characterization, data analysis, and manuscript drafting and editing. Z.S. contributed to sequence analysis and molecular characterization. N.C. contributed to molecular characterization. E.A. contributed to directed evolution. B.J.H. led project conceptualization, supervision, and manuscript editing.

## Conflicts of Interest

A.H. and B.J.H. have filed a patent application related to molecules described in this manuscript.

## Supporting information

Supporting File:

## Data Availability

The data that support the findings of this study are available from the corresponding author upon reasonable request.
