# Supplementary material for: Engineered B7‐H3 Binding in Modular Gp2 Miniproteins
Source: Biotechnol Bioeng. 2026 Apr 29;123(7):1871–81. doi: 10.1002/bit.70221 (PMC13245203; doi:10.1002/bit.70221)
Supplement: Supplementary file 1 — Supporting File: [file BIT-123-1871-s001.pdf]

Supplemental Information for:

Engineered B7-H3 binding in modular Gp2 miniproteins

Abbigael Harthorn[1], Hannah K. Windsor[2], Zachary Schmitz[2], Nathaniel Cheung[2], Ebube Agwaramgbo[2], and Benjamin J. Hackel[1,2]

Departments of [1]Biomedical Engineering and [2]Chemical Engineering and Materials Science
University of Minnesota – Twin Cities
Minneapolis, MN 55455

Correspondence: hackel@umn.edu

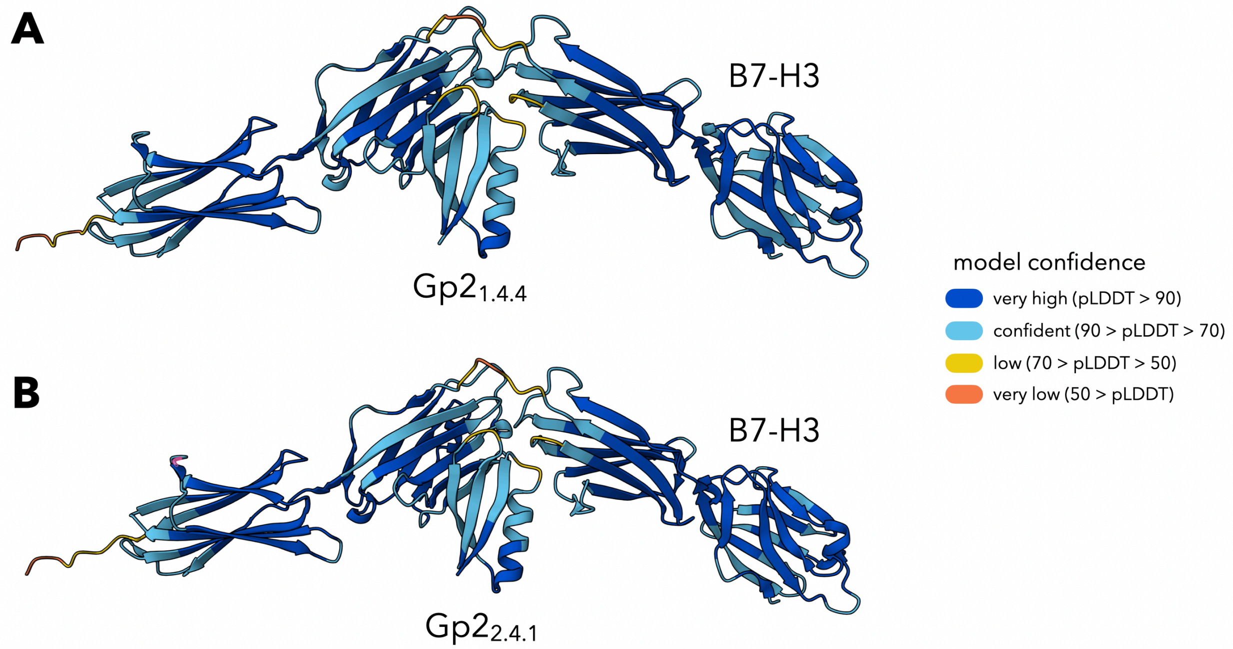

Figure S1. The complex between the extracellular domain of B7-H3 and $Gp2_{1.4.4}$ (A) or $Gp2_{2.4.1}$ (B) was predicted with AlphaFold3. Per-site pLDDT scores from AlphaFold3 are color coded.

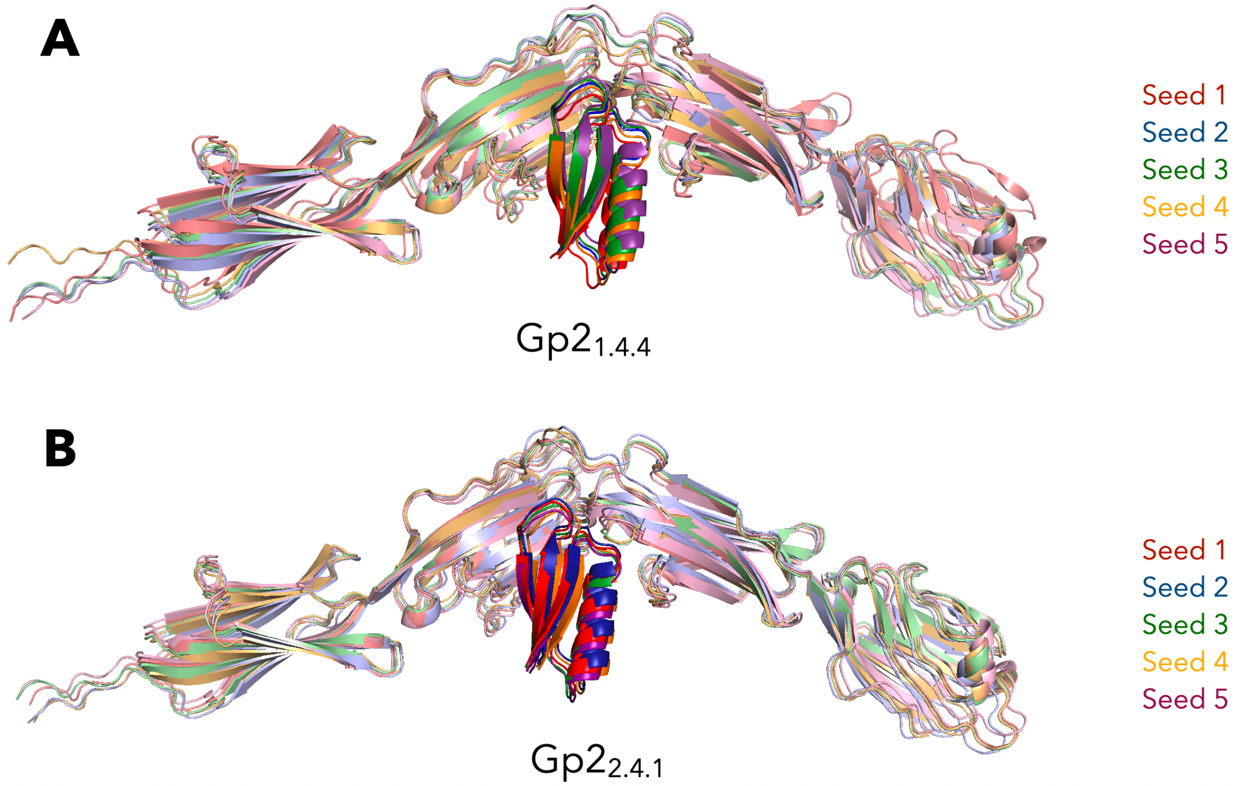

Figure S2. The complex between the extracellular domain of B7-H3 and Gp2$_{1.4.4}$ (A) or Gp2$_{2.4.1}$ (B) was predicted with AlphaFold3 for five independent seeds. The most confident pose for each of the five runs is presented.

Supplemental Table 1: Primers used for error-prone PCR

Primer Name	Sequence (5' -> 3')
W5	CGACGATTGAAGGTAGATACCCATACGACGTTCCAGACTACGCTCTGCAG
W3	GGATCCGAACAAAAGCTTATTTCTGAAGAGGACTTGTAATAGCTCGAGAT

Supplemental Table 2: Sequences for SortaseA$_{60-206}$ fusions

SortA$_{60-206}$ Sequence (5' -> 3')
CAAGCTAAACCTCAAATTCCGAAAGATAAATCAAAAGTGGCAGGCTATATTGAAAT
TCCAGATGCTGATATTAAAGAACCAGTATATCCAGGACCAGCAACACGCGAACAA
TTAAATAGAGGTGTAAGCTTTGCAAAAGAAAATGCATCACTAGATGATCAAAATAT
TTCAATTGCAGGACACACTTTCATTGACCGTCCGAACTATCAATTTACAAATCTTAA
AGCAGCCAAAAAAGGTAGTATGGTGTACTTTAAAGTTGGTAATGAAACACGTAAG
TATAAAATGACAAGTATAAGAAACGTTAAGCCAACAGCTGTAGAAGTTCTAGATGA
ACAAAAAGGTAAAGATAAACAATTAACATTAATTACTTGTGATGATTACAATGAAG
AGACAGGCGTTTGGGAAACCCGTAAAATCTTTGTAGCTACAGAAGTCAAA
